# The loopometer: a quantitative *in vivo* assay for DNA-looping proteins

**DOI:** 10.1093/nar/gkaa1284

**Published:** 2021-01-28

**Authors:** Nan Hao, Adrienne E Sullivan, Keith E Shearwin, Ian B Dodd

**Affiliations:** Department of Molecular and Biomedical Science, University of Adelaide, Adelaide, SA 5005, Australia; CSIRO Synthetic Biology Future Science Platform, Canberra, ACT 2601, Australia; Department of Molecular and Biomedical Science, University of Adelaide, Adelaide, SA 5005, Australia; Department of Molecular and Biomedical Science, University of Adelaide, Adelaide, SA 5005, Australia; Department of Molecular and Biomedical Science, University of Adelaide, Adelaide, SA 5005, Australia

## Abstract

Proteins that can bring together separate DNA sites, either on the same or on different DNA molecules, are critical for a variety of DNA-based processes. However, there are no general and technically simple assays to detect proteins capable of DNA looping *in vivo* nor to quantitate their *in vivo* looping efficiency. Here, we develop a quantitative *in vivo* assay for DNA-looping proteins in *Escherichia coli* that requires only basic DNA cloning techniques and a LacZ assay. The assay is based on loop assistance, where two binding sites for the candidate looping protein are inserted internally to a pair of operators for the *E. coli* LacI repressor. DNA looping between the sites shortens the effective distance between the *lac* operators, increasing LacI looping and strengthening its repression of a *lacZ* reporter gene. Analysis based on a general model for loop assistance enables quantitation of the strength of looping conferred by the protein and its binding sites. We use this ‘loopometer’ assay to measure DNA looping for a variety of bacterial and phage proteins.

## INTRODUCTION

Proteins that bind to specific DNA sites and are able to interact with each other to bring together separate DNA molecules or to form loops within the same DNA molecule are critical in essential DNA processes, such as transcription, replication, recombination and DNA organization (1–3). The primary role of the interaction can be to affect the function of the proteins at one or both sites. DNA looping provides cooperative binding over large distances, increasing site occupancy by the proteins (1,2,4), and can be used to target catalytic activities in transcriptional control, such as in activation of σ54 promoters (5) or eukaryotic enhancers (6,7). Bridging interactions between replicases bound to separate plasmids can inhibit replication initiation to provide plasmid copy number control (8,9). Alternatively, the primary role of the interaction can be the juxtaposition of the two DNA segments. Promoters can be repressed by being enclosed in small DNA loops (10,11), and the large chromatin loops formed by the interaction between insulator elements partition eukaryotic chromosomes into topological domains that are important for enhancer–promoter specificity (12). In many cases, the interaction combines both roles. Site-specific recombinases must juxtapose their DNA sites for correct recombination and the interactions in the synapsed complex also activate the recombinase catalytic steps (13). The long-range interaction between the *OL-* and *OR*-binding sites for the phage λ CI repressor increases CI binding at these sites, improving repression of the lytic promoters (14,15), while the DNA loop formed also juxtaposes a distal *UP* element near to the *PRM* promoter where it can activate lysogenic transcription (16,17).

Despite the importance of DNA looping and bridging, a general method for identifying proteins capable of bringing DNA together *in vivo* is not currently available. For most proteins, an *in vivo* looping capability is first implicated when the function of a protein at one binding site is affected by a distal binding site. For example, in the first described example of DNA looping by a transcription factor, looping by AraC was suspected because deletion of a binding site 270 bp upstream of the *araBAD* promoter affected AraC regulation of the promoter (18). To further support DNA looping, additional tests, usually specific to each system, are needed to rule out an independent function of the distal site. Helical phasing experiments, where the functional interaction between the sites is sensitive to insertion of nonintegral DNA turns in the intervening DNA, are often used to confirm DNA looping *in vivo* (18–21). However, helical phasing sensitivity is not present for long DNA loops (>500 bp), or for *trans* interactions, and may be minimized by protein flexibility. An alternative route to identifying DNA-looping proteins *in vivo* is provided by ligation-based proximity assays such as 3C (22). These techniques have revealed vast numbers of DNA loops in eukaryotic and bacterial genomes (23,24). However, additional approaches are needed to identify the proteins responsible for these loops. Thus, confirmation of the DNA looping or bridging activity of a specific protein often requires *in vitro* techniques such as DNA footprinting (25), electrophoretic mobility shift (26), enhancement of ligation (9) or single-molecule methods such as electron or atomic force microscopy (14,27) and tethered particle motion (28). However, these *in vitro* approaches need purified active protein and often require specialized expertise and equipment.

In addition, an important limitation of all these approaches is that they do not reveal the *in vivo* strength of the protein-mediated DNA looping, that is the fraction of time that the sites spend in direct contact in cells. Extraction of looping frequencies from functional *in vivo* assays has been possible only for highly characterized regulatory proteins, such as the Lac and CI repressors (29). Ligation-mediated proximity assays and techniques such as Dam-C can provide relative but not absolute frequencies of close proximity (30). Absolute proximity measures can be obtained by microscopic imaging of intact cells (31,32), but none of these proximity approaches is able to confirm direct protein-mediated contact. *In vitro* studies can quantitate looping; however, these measurements are made under conditions that may not reflect *in vivo* looping. Knowing how often the loops form *in vivo* provides critical information about the interplay between DNA looping and function, while identification of DNA-looping proteins capable of directing strong DNA looping will also aid in more effective manipulation of DNA looping in cells.

**Figure 1.**
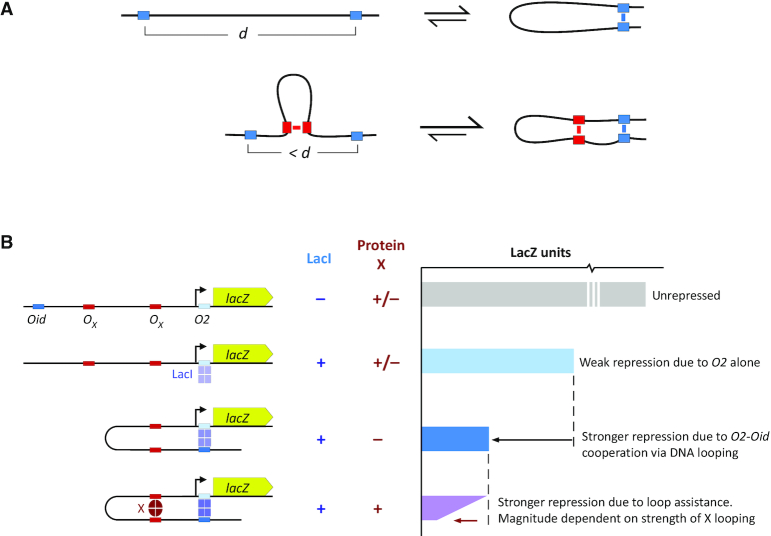
Assaying DNA looping by loop assistance. (**A**) The DNA loop formed between an internal pair of sites (red) assists the formation of the loop between the external sites (blue) by reducing the effective DNA distance, *d*, between them. (**B**) DNA looping by a candidate protein (X) and its DNA-binding sites (*O_X_*) is detected and measured in the loopometer by its enhancement of loop-dependent LacI repression of a promoter for a *lacZ* reporter. In the absence of the strong upstream *lacOid* operator, a LacI tetramer binds poorly to the weak *lacO2* operator, giving weak repression of the promoter. In the presence of *Oid*, occupation of *O2* is increased due to cooperative DNA looping, and repression is increased. DNA looping by the candidate protein shortens the distance between *Oid* and *O2*, increasing LacI looping and further improving repression.

Here, we report the development of an *in vivo* assay for DNA-looping proteins in *Escherichia coli* that does not depend on detailed knowledge of the protein’s function and requires only basic DNA cloning techniques and a LacZ assay. Furthermore, the assay provides a quantitative measurement of the strength of looping exerted by the protein and its binding sites. The assay is based on loop assistance, where the formation of one DNA loop assists the formation of another DNA loop (Figure 1A). When a pair of sites for one DNA-looping protein is nested within a pair of sites for another DNA-looping protein, DNA looping by the internal protein assists looping by the external protein by bringing its binding sites closer together (33–36). In the loopometer, the external loop is formed by two operators for the *E. coli* Lac repressor (LacI) that repress the expression of a *lacZ* reporter gene (Figure 1B). The proximal operator directly represses the promoter and the distal operator cooperates by DNA looping to increase LacI occupation of the proximal operator. This allows LacI looping to be detected and measured by the increase in repression in the presence of the distal operator (33,34). To assay DNA looping by a candidate protein, a pair of binding sites is inserted internal to the LacI loop and the protein is expressed. DNA looping by the candidate protein improves LacI looping by shortening the effective distance between the *lac* operators, detected by increased LacI repression of the reporter (Figure 1B). Analysis of the measurements using a general model for loop assistance (35) enables the looping strength of the protein and its binding sites to be estimated. We validate the assay using the λ CI protein and test a variety of bacterial and phage proteins for DNA-looping activity.

## MATERIALS AND METHODS

### General strains and media

All reporter strains are derivatives of E4643, itself a derivative of BW30270 MG1655 *rph*^+^ (CGSC7925) with the *lacIZYA* (MG1655:360,527–366,797) region removed (29). Plasmid replication of pLOM2–500 and pCYMR, which are derived from the CRIM plasmids of Haldimann and Wanner (37), is dependent on the R6Kγ π replicase protein Pir. We thus maintain these plasmids in the Pir-expressing (*pir^+^*) strain E4644 = EC100D (Epicentre) = F^–^*mcrA* Δ(*mrr-hsdRMS-mcrBC*) φ80d(*lacZΔM15*) Δ*lacX74 recA1 endA1 araD139 Δ(ara-leu)7697 galU galK* λ^–^*rpsL* Str^R^*nupG pir^+^*(DHFR).

LB (1% Bacto-tryptone, 1% NaCl, and 0.5% yeast extract, pH 7.0) is used for routine growth of strains. M9MM-glyc = 1 × M9 salts, 2 mM MgSO_4_, 0.1 mM CaCl_2_, 0.01 mM (NH_4_)_2_Fe(SO_4_)_2_·6H_2_O, and 0.4% glycerol [10 × M9 salts = 67.8 g of NaH_2_PO_4_, 30.0 g of KH_2_PO_4_, 10 g NH_4_Cl and 5 g NaCl/l H_2_O] were used for LacZ assays. Antibiotics used were (μg/ml for selection of integrated copy/plasmid copies): Ap, ampicillin (-/100); Cm, chloramphenicol (20/30); Km, kanamycin (20/50); Sp, spectinomycin (20/50); Tc, tetracycline (3/20). A 100 mM stock of cumic acid (CA; Sigma-Aldrich Cat. 268402) was prepared in ethanol.

### Constructing loopometer strains

#### Cloning protein binding sites into pLOM2-500

**Figure 2.**
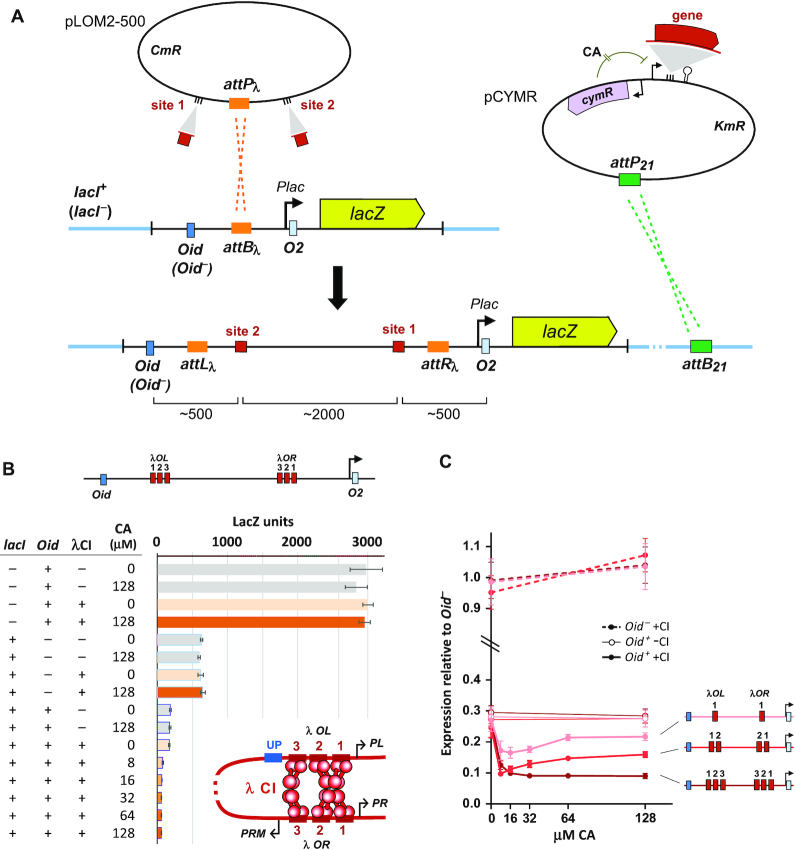
The loopometer system. (**A**) The basic procedure for the construction of the looping reporter strains. Protein-binding sites are cloned into the pLOM2–500 plasmid and the resulting plasmid is inserted by λ integrase into a reporter landing pad in the *Escherichia coli* chromosome, causing the two sites to be nested inside a pair of *lac* operators, *Oid* and *O2*, which control the *PlacUV5* promoter for a *lacZ.O2^–^* reporter gene. LacI is supplied by a *PlacI.lacI^+^* gene inserted elsewhere in the chromosome. The pLOM2–500 plasmid is also integrated into control strains lacking *lacI^+^* or *Oid*. The gene for the candidate protein is cloned into the pCYMR expression vector, under the control of the CymR repressor that can be inactivated by cumic acid (CA). This expression vector or the empty control is inserted by φ21 integrase-mediated recombination into the chromosome of the three strains carrying pLOM2–500. (**B**) LacZ assay results for λ CI and λ *OL123*-*OR123*. DNA looping is indicated by a LacI-dependent, Oid-dependent, candidate protein-dependent decrease in units. Error bars are 95% confidence limits (Student's *t*), *n*= 6. The insert shows DNA looping by a CI octamer and tetramer at the native *OL* and *OR* sites. (**C**) Graphic display of results for λ CI and *OL123-OR123*, *OL12-OR12*, *OL1-OR1* reporters (*lacI^+^* only). LacZ units within each set are normalized to the average of the four *Oid^–^* values (±CI, ±CA).

The restriction sites flanking sites 1 and 2 in pLOM2–500 (Figure 2A and Supplementary Figure S1A) allow various strategies for cloning candidate binding sites at these positions. We use Gibson isothermal assembly (38), either by 2-fragment assemblies with a single DNA fragment comprising both inserts and *attP_λ_* (PCR-generated or purchased commercially) or by 4-fragment assemblies of two polymerase chain reaction (PCR)-generated insert fragments, an *attP_λ_* fragment and digested and isolated plasmid backbone. pLOM2–500 plasmids were maintained in E4644 (LB + Cm, 30 μg/ml). Supplementary Figure S2B shows the detailed structure of pLOM2–500 and the reporter after its integration into the loopometer landing pad. Primers #2238 (TGGCGACGCTCATGTATGTG) and #2239 (CTCTTACGTGCCGGAAGT) were used for sequencing the site 1–site 2 region.

#### Cloning protein genes into pCYMR

The pCYMR plasmids (Figure 2A and Supplementary Figure S3A) contain a multiple cloning site (SpeI.ApaL1.XmaI.PvuII.AflII.AccIII.NarI) located downstream of the CymR-binding site (*cymO*), followed by transcription terminators. We generally used 2-fragment Gibson assembly to insert PCR-generated fragments of protein genes into the PvuII site. Sequences for translation of the protein message need to be included in the insert. pCYMR plasmids were maintained in E4644 (LB + Km, 50 μg/ml). Inserted sequences were confirmed using primers #2037 (CATGATCACCATAGATCCTTTCTCC) and #787 (ACCGGTTAATTAACGGCACCACCGAA). Three variants of pCYMR were made with altered activity of the expression promoter: pCYMR.1 (standard vector), pCYMR.4 (higher expression) and pCYMR.6 (lower expression; Supplementary Figure S3). The detailed structure of the pCYMR expression plasmids and the cloning junctions for the genes inserted are given in Supplementary Figures S3 and S4.

#### Integrating pLOM-500

The pLOM-500 reporter plasmid containing the protein-binding sites is separately integrated into three strains: AH6112 (*lacI^–^Oid^+^*), AH6113 (*lacI^+^Oid^–^*) and AH6114 (*lacI^+^Oid^+^*). These strains differ in whether *Oid* is present at the distal site in the loopometer landing pad (*Oid^+^*) or absent (*Oid^–^*; Figure 2, sequences in Supplementary Figure S2D), and whether the pIT3-SH.lacI-rev plasmid (*lacI^+^*; Supplementary Figure S2E) or the empty vector pIT3-SH (*lacI^–^*) is integrated at *attP_HK022_*. Strains AH6112, AH6113 and AH6114 also contain the pINTts λ Int helper plasmid (37), which express λ Int at temperatures above 30°C and carry a temperature-sensitive pSC101 *repA101* gene needed for its maintenance. For transformation and integration, these cells are grown at 30°C in LB + Ap, 100 μg/ml to OD_600_ ∼0.2–0.4, transferred to 39°C for 20 min, before being made competent and transformed with pLOM-500 vectors by the TSS method (39), with selection for integrants on LB + Cm, 20 μg/ml plates at 37°C. Purified colonies are tested for correct pLOM-500 integration by PCR (40) with a mix of primers #462 (ATGACAGAGGCAGGGAGTGG), #1729 (TTGTGCTTCTCTGGAGTGCG), #1685 (GTATCCCCACTCACTTAGTC) and #1686 (CCAGTGAATCCGTAATCATGG), which give 351, 841, 470 and 727 bp *attP*, *attB*, *attL* and *attR* fragments, respectively.

#### Integrating pCYMR

The three resulting strains are then transformed with the pAH121 φ21 Int helper plasmid (37), with outgrowth in LB at 30°C and selection on LB + Ap, 100 μg/ml plates at 30°C. Integration competent cells of these strains are then made and transformed (as above) with empty pCYMR or the pCYMR plasmid containing the candidate protein gene, with selection on LB + Km, 20 μg/ml plates at 37°C. Purified colonies are tested for correct integration by PCR (40) with a mix of primers #465 (GGGAATTAATTCTTGAAGACG), #467 (ACTTAACGGCTGACATGG), #871 (ATCGCCTGTATGAACCTG) and #2404 (GCAGCCTAACAAAAAACAACG), which give 1226, 420, 621 and 1025 bp *attP*, *attB*, *attL* and *attR* fragments, respectively. Alternative methods for expressing the candidate protein may be used, as long as they do not require selection for chloramphenicol (the reporter module) or spectinomycin (the LacI expression module), and are not under LacI control.

### LacZ assays

The standard loopometer assay involves LacZ (β-galactosidase) assay of six strains, all with the same protein-binding sites inserted at loopometer sites 1 and 2. The strains have three different combinations of *lacI* and *Oid*: *lacI^–^Oid^+^*, *lacI^+^Oid^–^* and *lacI^+^Oid^+^*, and carry either the integrated empty pCYMR vector or the pCYMR vector expressing the relevant protein.

Strains were grown and kinetic LacZ assays were performed in 96-well flat-bottomed microtiter plates. Six fresh colonies of each strain on LB + Cm, 20 μg/ml plates (except for ID1292 and ID1293, which were selected with Km20) were picked with a 1000 μl micropipette tip and resuspended in 40 μl M9MM + Cm20. About 5 μl of each resuspension was added to 95 μl M9MM+ Cm, 20 μg/ml + CA in a microtiter plate, sealed and incubated with shaking at 37°C overnight. These cultures were then diluted 5 μl into 95 μl fresh medium and incubated for ∼3 h. The five control strains were grown in 0 and 128 μM CA, while the *lacI^+^ Oid^+^* (pCYMR-X) strain was grown with 0, 8, 16, 32, 64 and 128 μM CA (see Figure 2B).

Cultures were grown to OD_600_ 0.3–0.8, measured using a Labsystems Multiskan Ascent plate reader with a 620 nm filter. The OD_620_ values were converted to OD_600_ (1 cm path length) values using an empirically derived relationship. For the assay, 5 μl of culture was added to 195 μl warmed assay buffer in a fresh microtiter plate, consisting of 88 μl TZ8 buffer, 60 μl 4 mg/ml o-nitrophenyl-β-D galactoside (Sigma-Aldrich Cat. N1127) in TZ8, 2 μl 10 mg/ml chicken egg white lysozyme (Sigma-Aldrich Cat. L6876, 40,000 units/mg) in TZ8, 4 μl 20 mg/ml polymyxin B (Sigma-Aldrich Cat. *P*-4932) in H_2_O, 6 μl H_2_O and 35 μl M9MM. TZ8 buffer is 100 mM Tris-HCl, pH 8.0, 1 mM MgSO_4_, 10 mM KCl. The plate was incubated at 28°C in the plate reader, with OD_414_ readings taken every 2 min for 1 h. Enzyme activity was determined as the slope of the line of best fit of OD_414_ versus time (readings with OD_414_ > 2.5 were ignored). LacZ units were calculated as 200 000 × (OD_414_/min)/(OD_600_ x 5 μl). The improved linearity compared with our previous assay (41) is examined in Supplementary Figure S5.

### Quantitation of looping

*F_L(X)_*, the fractional LacI looping in the presence of the candidate protein, is obtained from the LacZ activities of the *lacI^+^Oid^+^* and *lacI^+^Oid^–^* reporters with the expression of the protein, combined with the background LacZ activity of strain ID1285, using Equation (1) (Figure 4A).

Estimation of *I*_L_/*I*_X_ from this *F_L(X)_* value uses Equation (2) (Figure 5B), which requires additional *F_L_* measurements to estimate the weights *p*, *q* and *r*. The derivation of Equation (2) is based on our statistical-mechanical model for loop assistance (Figure 5; 35). The weight *p* is obtained from the *F_L_* measurement of LacI looping of the *lacI^+^Oid^+^* and *lacI^+^Oid^–^* versions of the loopometer in the presence of empty pCYMR. The weight *q* is estimated from assays of the *lacI^+^Oid^+^* and *lacI^+^Oid^–^* strains ID1290 and ID1291, in which *Oid* and *Plac.O2* are separated by a b’ sequence similar to that between sites 1 and 2 (b, Figure 5B). The weight *r* is estimated from assays of the *lacI^+^Oid^+^* and *lacI^+^Oid^–^* strains ID1292 and ID1293, in which *Oid* and *Plac.O2* are separated by a concatenation of the 500 bp *Oid*-site 1 and site 2-*Plac.O2* a and c arms separated by a 45 bp λ *OR21* segment (Figure 5B).

### Availability

Plasmids pAS0007 ( = pLOM2–500 with φKO2 operators: Addgene # 164868), pID1072 ( = pCYMR-1 with φKO2 cB: Addgene # 164869) and plasmid pAH121 (Addgene #164901), as well as strains AH6112–4, ID1285, and ID1290–3 will be deposited in Addgene.

### Details of system construction

#### pLOM plasmids

Plasmid pLOM1–500 is an earlier version of pLOM2–500 that does not carry flanking restriction sites on both sides of sites 1 and 2, and was used for reporters with some binding sites (Supplementary Figure S1B). The sequences of protein binding sites cloned into these vectors are listed in Supplementary Figure S1.

#### pCYMR plasmids

The CymR repressed promoter in these vectors was derived from the T5 promoter.*cymO* sequence of pNEW (42), which includes an unwanted overlapping *lac* operator and also displayed leaky CymR repression. We randomized the sequences between the -35 and -10 hexamers (including the first -10 basepair) to remove this operator and alter promoter activity to generate the pCYMR-1, pCYMR-4 and pCYMR-6 variants (Supplementary Figure S3).

#### Loopometer recipient strains

The reporter landing pad present in strains AH6112, AH6113 and AH6114 was constructed in two steps by recombineering (Supplementary Figure S2A) using pSIM6 (43). First, a PCR fragment containing a kanamycin resistance gene (*KmR*) and a distal portion of the *lacZ* gene was used to replace *attB_λ_* in the E4643 chromosome to give strain AH6101 (Supplementary Figure S2A). Second, after reintroduction of pSIM6, a PCR fragment carrying *Oid^+^* (or *Oid^–^*), *attB_λ_*, *PlacUV5.lacO2* and the proximal portion of the *lacZ(O2^–^)* gene (Supplementary Figure S2A) was used to replace *KmR*, with screening for *lacZ^+^* on LB + X-gal (20 μg/ml) plates. These two strains were transformed with the pAH69 HK022 Int helper plasmid (37) and pIT3-SH.lacI-rev (Supplementary Figure S2E), or the empty vector pIT3-SH were then integrated at *attP_HK022_*. The resulting strains were transformed with the pINTts λ integrase helper plasmid (37) to give AH6112 (*lacI^–^Oid^+^*), AH6113 (*lacI^+^Oid^–^*) and AH6114 (*lacI^+^Oid^+^*).

#### ΔdeoR strains

Strains equivalent to AH6112 (*lacI^–^Oid^+^*) and AH6114 (*lacI^+^Oid^+^*) but carrying a deletion of the *deoR* gene were made by recombineering, first replacing sequences between MG1655:CATCAACTTAATGCG 881996 and 882732 ATAATCCCTCTGAA with a *KmR* cassette flanked by FRT sites, followed by FLP-mediated removal of the *KmR* gene using the pE.FLP plasmid (40), resulting in an in-frame deletion of almost the entire *deoR* gene.

#### The Plac^–^ strain for measuring reporter background

A DNA segment spanning the leftward *FRT* site, chloramphenicol resistance gene (*CmR*) and *PlacUV5* was obtained from AH6114 carrying an integrated pLOM2–500 vector (Supplementary Figure S2B) and cloned into a plasmid, where mutations were introduced into the –35 and –10 sequences of *PlacUV5* (Supplementary Figure S2D). A PCR fragment (Supplementary Figure S2C) from this *Plac^–^*plasmid was used for recombineering into an AH6114 (pLOM2–500) strain in which the sequences between the FRT sites (Supplementary Figure S2B) had been removed by treatment with pE.FLP (40), allowing selection for the *CmR* gene in the insert. The resulting *Plac^–^*strain, ID1285 is *lacI^+^Oid^+^*, with *matS* null sequences (Supplementary Figure S1) at sites 1 and 2.

#### Strains for calibration of LacI looping

The DNA sequences for construction of b’ and ac strains (Supplementary Figure S6) were assembled in plasmids, which were then used to generate PCR fragments (Supplementary Figure S6) for recombineering into the chromosome of AH6101 (Supplementary Figure S2A), with selection for *CmR* (b’) or screening for *lacZ^+^* (ac). Successive integration of pIT3-SH.lacI-rev and empty pCYMR gave b’ strains ID1290/ID1291 (*Oid^+^/Oid^–^*) and ac strains ID1292/ID1293 (*Oid^+^/Oid^–^*). The b’ segment shares 1870 bp with the loopometer b segment, with divergent sequences at each end near *Oid* and *Plac.O2*. The ac strain carries a 45 bp λ *OR21* insert between the fused sites 1 and 2.

Extended reporters were made by insertion of plasmid pID1302 into the *attB_3_* site of the b’ reporters ID1290/ID1291, and full-length *lacI^+^Oid^+^*reporters (abc) carrying the *matS* null site (Supplementary Figure S1) at both sites 1 and 2. Plasmid pID1302 (Supplementary Figure S6B) carries the *attP_3_* site, the *pir*-dependent R6Kγ origin, a tetracycline resistance gene (*TcR*) and ∼6 kb of spacer sequence derived from the *E. coli ftsK* and *rne* genes. The *int3* gene and the Int3 attachment sites are from the collection of Yang *et al.* (44). Integration of pID1302 (Supplementary Figure S6B) was mediated by expression of Int3 from the helper plasmid pAH6046, which was derived from pINTts (37) by replacing λ *int* with the *int3* gene.

Sequences of strains and plasmids are available on request.

## RESULTS AND DISCUSSION

### Procedure for testing DNA looping by a candidate protein

The standard assay uses a looping reporter and a separate module for the controlled expression of the protein, both of which are integrated into the bacterial chromosome (Figure 2A).

The looping reporters are constructed by insertion of two potential binding sites for the candidate protein at sites 1 and 2 on either side of the bacteriophage λ *attP* attachment site (*attP_λ_*) in plasmid pLOM2–500, using standard cloning techniques (‘Materials and Methods’ section). The resulting plasmid is then integrated into the chromosome of three different *E. coli* strains by recombination into an *attB_λ_* site on a specially constructed ‘landing pad’ (Figure 2A). Integration is catalyzed by λ integrase expressed from a separate helper plasmid (37). Integration results in the two binding sites being located ∼2 kb apart, separated by the inactive plasmid replication origin and the chloramphenicol resistance gene, with the proximal binding site located ∼500 bp upstream of a *lacO2* operator controlling a promoter (*PlacUV5*) for a *lacZ* reporter gene. Integration into strain AH6114 produces the intact loop reporter, which has a second *lac* operator, *Oid*, located ∼500 bp upstream of the distal binding site, and a *lacI* gene elsewhere on the chromosome. The AH6112 and AH6113 strains lack either *lacI* or *Oid*, respectively, and serve as controls.

For expression of the candidate looping protein, we routinely use a chemically inducible expression system based on the CymR repressor from *Pseudomonas putida* F1 (42), which is inactivated by added CA. This allows testing of a range of protein concentrations, which may be important if high protein levels give submaximal looping, as seen for LacI (29), or are toxic. The gene for the candidate looping protein is inserted into the pCYMR plasmid, and the resulting plasmid is transformed into the three reporter strains previously transformed with the phage φ21 integrase helper plasmid (37), for catalyzing integration at the φ21 attachment site on the bacterial chromosome (*attB_21_*) (Figure 2A). Our standard pCYMR.1 vector gives a low level of uninduced ‘leak’, with strong induction by CA (Supplementary Figure S3B). Nevertheless, we generally also integrate an empty pCYMR.1 plasmid into the reporter strains to provide control strains with no candidate protein.

Once the strains have been constructed, LacZ assays are done to test whether CA-induced expression of the candidate protein gives an *Oid*-dependent increase in LacI repression of the *lacZ* gene (Figure 1B). We use a microtiter plate-based version of the basic Miller LacZ assay (‘Materials and Methods’ section); however, standard Miller assays are adequate.

### Validation of the assay with λ CI

We tested the assay with the λ CI repressor (45). Binding of CI dimers to individual operators via the N-terminal domains, and further association of these dimers to tetramers and octamers mediated by the C-terminal domain (46,47), is able to produce a variety of DNA loops. The natural cooperative binding of two CI dimers to pairs of adjacent operators at its *OR-* and *OL*-binding sites is due to mini-loops, with the operators, which are spaced ∼2 DNA turns apart, being ‘looped’ by a CI tetramer (17,48). DNA looping has also been seen between single operators spaced 5 or 6 DNA turns apart (25). Longer DNA loops—beyond 2 kb *in vitro* and *in vivo*—have been observed for the interaction of two tetramer-bound sites, resulting in four operators linked by a CI octamer (14,17,28). The natural three-operator groupings at *OL* and *OR* are capable of even stronger looping (28,35), presumably because the two operators not bound by the octamer are bridged by an additional CI tetramer (49) (Figure 2B). We have shown that the full *OL* and *OR* sequences can form a 20 kb loop with ∼16% efficiency *in vivo* (35).

Figure 2B shows the results of CA-controlled expression of λ CI protein with λ *OR* and *OL* inserted at sites 1 and 2 in the reporter. In the *lacI^–^* background, the reporter is unrepressed, giving ∼3000 LacZ units, and this activity is unaffected by the CI expression module, with or without full induction by CA (128 μM). In the *lacI^+^* background but in the absence of the upstream *Oid* operator, the reporter is repressed ∼5-fold due to weak binding of LacI to the proximal *O2* site. Again, this activity is unaffected by CI expression. These controls check that any binding of the candidate protein to its sites does not directly affect the promoter or affect LacI repression in the absence of the upstream *Oid*. In the presence of *Oid* and the absence of CI, LacI repression of the reporter increases ∼4-fold. This loop-dependent repression effect is due to the strong *Oid* site binding a LacI tetramer and thus fixing the free DNA-binding domain of the tetramer at the end of a 3 kb tether attached to *O2*. At this distance, the effective concentration of the LacI DNA-binding domain seen by *O2* is substantially higher than the concentration of the LacI DNA-binding domains of free LacI tetramers, resulting in frequent DNA looping and increased occupation of *O2*.

In the presence of CI, repression in the *lacI^+^Oid*^+^ reporter is increased even further (Figure 2B). Given the results of the control strains, this increased repression can be interpreted as DNA looping by CI between *OL* and *OR* that, at least some of the time, shortens the distance between *Oid* and *O2* to increase their relative concentration and increase LacI looping (Figure 1).

We generally test a range of induction levels for the candidate protein, as DNA looping generally has a concentration optimum (29,34). Too low protein concentration gives insufficient occupation of the DNA sites; too high concentration causes loop blockage, where the sites are occupied by higher order multimers that are unable to interact further (e.g. LacI tetramers at both *lac* operators).

To test whether the assay could display differences in the strength of looping, we compared λ CI looping of the native 3-operator *OL* and *OR* sites with looping by 2-operator or single operator sites. The equivalent assay to Figure 2B was done for reporters with these sites, and a subset of the results for all three combinations is plotted in Figure 2C. These plots are normalized to the average units obtained for each *Oid*^–^ reporter (± CI, 0/128 μM CA) to account for day-to-day variation in the LacZ assay.

As expected, the single operator *OL1-OR1* reporter displayed weaker looping than the multioperator combinations, with CI expression giving less assistance to LacI repression at all induction levels. We note that this is the first time that looping has been observed for single CI operators spaced more than a few DNA turns apart. Looping by the 2-operator *OL12-OR12* combination was also weaker than the 3-operator pair at most CI induction levels.

Interestingly, the 1- and 2-operator combinations gave maximal looping at low CI and less looping as CI levels increased. Formally, this can be explained as being due to the formation of higher order looping-incompetent CI multimers at each site. However, we were surprised to see this effect for λ CI, as it implies that CI octamers are forming independently at 1- or 2-operator sites. This effect was not seen for the 3-operator *OL* and *OR* sites, presumably because loop blockage is counterbalanced by loop-promoting bridging between the third operators (49).

Having validated the assay, we then used it to test other known or suspected DNA-looping proteins.

### DeoR

The *E. coli* DeoR protein utilizes DNA looping in its repression of an operon for catabolism of nucleosides. DeoR binds to single operators (O_1_ and O_2_) spaced ∼600 bp apart at each of a pair of tandem promoters, with a third site (O_E_) located ∼300 bp further upstream. Repression at either promoter is improved in the presence of the other sites and is detectable when O_1_ is placed ∼5 kb away from O_2_ (50). DNA loops between all sites have been observed by electron microscopy (51).

**Figure 3.**
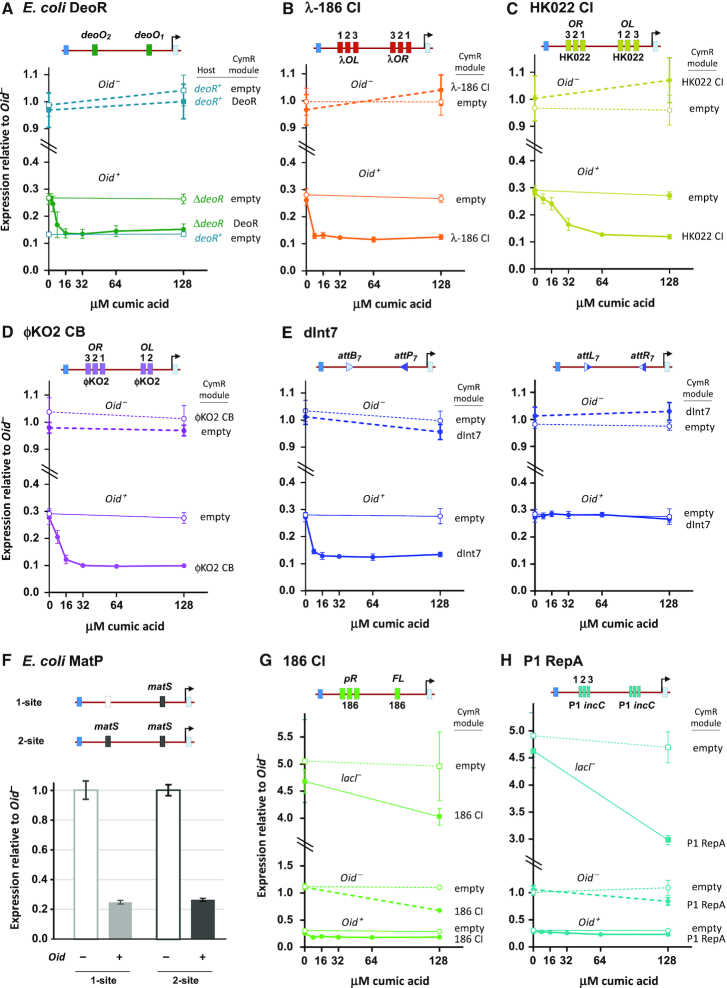
Testing candidate proteins and sites for DNA looping. LacZ units produced by loopometer reporters in *lacI^+^Oid^+^* and *lacI^+^Oid^–^* strains, normalized to the average of the *Oid^–^* values. Protein expression was induced with CA from chromosomally integrated pCYMR vectors or an empty vector control. Measurements in *lacI^–^Oid^+^* strains showed no substantial effects of candidate proteins in the absence of LacI and are omitted to save space, except for (G) and (H). Error bars are 95% confidence limits (Student's *t*), *n* = 6 unless otherwise stated. Sequences of inserts at loopometer sites 1 and 2 are given in Supplementary Figure S1; protein sequences inserted in pCYMR are given in Supplementary Figure S4. (**A**) DNA looping between *deoO_1_* and *deoO_2_* operators by endogenous or exogenous *E. coli* DeoR. (**B**) DNA looping between full λ *OL123* and *OR123* sites by a hybrid protein with the λ CI DNA-binding N-terminal domain and the phage 186 CI C-terminal association domain. (**C**) DNA looping between phage HK022 *OL* and *OR* by HK022 CI. (**D**) DNA looping between phage φKO2 *OL* and *OR* by φKO2 CB. (**E**) DNA looping by integrase dInt7 (catalytically inactivated) between its *attB* and *attP* sites (left), but a lack of looping between its *attL* and *attR* sites (right). (**F**) A lack of looping between *matS-*binding sites by endogenous *E. coli* MatP is indicated by the absence of decreased LacZ units compared to a control reporter with only a single *matS* site. (**G** and **H**) Inconclusive looping assays for phage 186 CI looping between its *pR* sites and regulatory *FL* site, and for phage P1 RepA looping between P1 triple-iteron sites. In both cases, the expressed proteins have confounding *lacI*-independent and *Oid*-independent effects.

We inserted the deoO_1_ and O_2_ operators into sites 1 and 2 of the loopometer reporter, the *deoR* gene into the pCYMR-1 expression module and tested loop assistance in strains in which the endogenous *deoR* gene was deleted (‘Materials and Methods’ section). A clear *Oid*-dependent increase in LacI repression was seen with increasing expression of DeoR (Figure 3A). A similar degree of looping was seen for the reporter exposed to the endogenous level of DeoR protein in a *deoR^+^* strain (Figure 3A).

### Hybrid λ-186 CI repressor

We have previously shown that a hybrid repressor in which the λ CI DNA-binding N-terminal domain (NTD) is fused to the phage 186 CI oligomerization C-terminal domain (CTD) is able to regulate the λ *PR* and *PRM* promoters at *OR* in a way that responds to the presence of *OL* located 3.8 kb away (52). The intact 186 CI protein is a nonlambdoid repressor that uses distal binding sites for regulation *in vivo* (53) and forms DNA loops *in vitro* (27). The 186 CI CTD forms a wheel-like 14-mer structure that should present attached NTDs on its rim for DNA binding (52). It is thus very likely that this hybrid protein can form DNA loops; however, this has not been independently confirmed.

When we expressed the λ-186 CI hybrid from integrated pCYMR-1 (‘Materials and Methods’ section) in the full λ *OL-OR* loopometer reporter, we saw an increase in *Oid*-dependent LacI repression indicative of DNA looping (Figure 3B). Looping was weaker than we saw with intact λ CI (Figure 2C).

### Lambdoid phage repressors: HK022 CI and φKO2 CB

φHK022 is a lambdoid phage with a similar basic genomic arrangement to λ, including divergent lytic operons controlled by *OL* and *OR* elements separated by the immunity region, including the *cI* gene (54). The natural distance between *OL* and *OR* is ∼700 bp in HK022, compared to 2.3 kb in λ. The HK022 CI repressor shows homology with other lambdoid repressors and is known to bind cooperatively to adjacent operators within *OR* and *OL* (54). DNA looping by HK022 CI seems possible given these similarities to λ, and is further suggested by loop-like DNase I sensitivities induced by CI in the 53 bp between *OL2* and *OL3*, and by the location of a CI operator 300 bp downstream of *OR* that is involved in immunity (54,55). However, DNA looping has not been tested.

The entire HK022 *OR* and *OL* regions (with mutations to inactivate the *pL* and *pR* promoters; Supplementary Figure S1A) were inserted at the loopometer sites 1 and 2 (Supplementary Figure S2). Expression of HK022 CI produced an Oid-dependent increase in LacI repression of the reporter, clearly showing DNA looping (Figure 3C).

*Klebsiella oxytoca* phage φKO2 is another lambdoid phage, closely related to N15. These ‘telomeric’ phages are unusual in forming nonintegrated linear prophages and are classed as lambdoid based on their operon structures and sequence similarities (56). The φKO2 immunity repressor CB shows homology with lambdoid repressors and represses lytic transcription from *OL* and *OR* regions located ∼630 bp apart, either side of the *cB* gene (57). The similarities to λ led Hammerl *et al.* (57) to speculate that φKO2 CB protein might form a DNA loop between *OL* and *OR*. However, this has not been experimentally validated.

We inserted φKO2 *OL* and *OR* (with mutations to inactivate promoters; Supplementary Figure S1A) into the loopometer and expressed the φKO2 CB protein. Increased LacI repression seen in the presence of CB induction confirmed *OL-OR* looping by CB (Figure 3D).

Thus, all three of the lambdoid phage repressors tested—λ, HK022 and φKO2—display DNA looping between the operators that control early lytic transcription. The genomic arrangement of divergent lytic promoters separated by a short immunity region containing the repressor gene is a module common to many lambdoid phages, suggesting that long-range repressor looping is widespread. In λ, CI looping increases repression of the lytic promoters (14,15), and also provides complex control of the immunity promoter (17). Looping-dependent cooperative repression of the lytic promoters seems likely to be a shared feature; however, differences in the operator arrangements in HK022 and φKO2 suggest that these and other lambdoid phages may use looping differently in control of the immunity promoter.

### dInt7—an inactivated serine integrase

Site-specific recombination between sites either on the same or different DNA molecules must involve the formation of a protein bridge between the two DNA sites. However, the strength of this DNA bridging capability is not clear, since the catalytic steps of recombination may be sufficiently fast that only a transient DNA contact is required.

Serine integrases are a large class of site-specific recombinases that, in the absence of a recombination directionality factor (RDF), catalyze a unidirectional reaction between their attachment sites—attP + attB → attL + attR—without the need of cofactors such as IHF (58). The bridging complex is an Int tetramer with the two DNA sites each bound to an Int dimer.

To test the DNA-looping capacity of serine integrases, we used Int7, one of a set of 34 large serine integrases isolated and characterized by Yang *et al.* (44). We made an inactive variant—dInt7—by mutating the catalytic serine (Ser10) to alanine, and inserted this into the pCYMR-1 expression module. The cognate *attB_7_* and *attP_7_* sites were inserted into sites 1 and 2 of the loopometer reporter, in an inverted orientation. We saw a clear *Oid*-dependent increase in LacI repression when dInt7 was expressed (Figure 3E), indicative of substantial DNA looping between *attB_7_* and *attP_7_*.

The inability of the serine integrases to recombine *attL* and *attR* is thought to be due to poor synapsis between Int-bound *attL* and *attR* sites. Electrophoretic mobility shift assays with φC31 and BxB1 integrases found synaptic complexes only between *attP* and *attB* (59,60). A model stimulated by structural studies of the LI integrase bound to *attP* DNA suggests that upon completion of the DNA strand cleavage and rejoining reactions between *attP* and *attB*, new *cis* interactions form between the Int monomers bound at *attL* and *attR* that prevent Int tetramerization (61).

To test the expected lack of synapsis between the *attL_7_* and *attR_7_* sites, we used transient expression of active Int7 to recombine *attP_7_* and *attB_7_* in the loopometer reporter, generating reporters with an inverted 2 kb internal segment flanked by *attL_7_* and *attR_7_* sites. Expression of dInt7 did not result in detectable DNA looping in these reporters (Figure 3E). This result provides *in vivo* confirmation of an inability of the integrase alone to bring *attL* and *attR* together, at least for Int7. However, the assay does not by itself show whether the looping defect is due to a lack of Int binding to *attL* or *attR* or whether Int is bound but cannot loop.

In general, it is not possible to be certain from a negative loopometer result that a protein and sites are incapable of looping, because the assay does not provide independent confirmation that the protein is expressed, is active and is binding its DNA sites. In the case of dInt7, we at least know that the expressed protein is active by its looping of *attP_7_*-*attB_7_*, but while other serine integrases are known to bind to *attL* and *attR* sites *in vitro* (59,60), it is possible that this binding is not occurring for dInt7 *in vivo*.

### A negative result for MatP:matS

Another example of a negative result is provided by our examination of looping by MatP. The *E. coli* MatP protein binds to 13 bp sites termed *matS* that are clustered around the replication terminus of the chromosome. Some 23 *matS* sites are distributed over an ∼800 kb region corresponding to the Ter macrodomain, with MatP and *matS* sites functioning to structure and insulate this domain (62). X-ray crystallography of MatP–*matS* complexes showed a MatP tetramer forming a bridge between two separate *matS* DNAs, with DNA looping also observed *in vitro* by electron and atomic force microscopy (63), leading to the idea that the Ter domain might involve a network of MatP–*matS* looping. However, more recent Hi-C measurements did not reveal strong contacts between *matS* sites. This and other results indicate that MatP exerts its effects without DNA looping (64).

To test MatP/*matS* looping, we constructed loopometer reporters carrying a *matS* sequence at sites 1 and 2. To avoid effects on the cells due to the elimination of the endogenous MatP protein (62), we made control reporters without *matS* at site 1. We found that LacI looping was not enhanced by the presence of the second *matS* site, indicating a lack of looping (Figure 3F).

This result supports the idea that MatP does not loop *matS* sites *in vivo*. However, our assay does not confirm the binding of MatP to our *matS* sites, and it is possible that the DNA sequence context or the cellular location somehow reduces binding at our sites relative to the natural sites at Ter. The conflict between the *in vitro* evidence for looping and the *in vivo* results may be due to a variety of factors. The *in vivo* concentration of MatP may be far from optimal for looping; either too low to give significant occupancy of *matS* sites (note that ChIP assays (62) can be positive even when occupancy is low) or so high that MatP tetramers bind independently to each *matS* site. Bound MatP dimers may also interact with other proteins that block dimer–dimer contacts *in vivo*.

### Inconclusive results: 186 CI and P1 RepA

In two cases, testing of DNA looping in the loopometer gave inconclusive results because the expression of the candidate protein affected LacZ units in the control strains lacking either LacI or *Oid*.

In attempting to test DNA looping by intact phage 186 CI repressor between its *FL* and *pR* regulatory sites (53), we found that while expression of 186 CI reduced LacZ units in the *lacI^+^Oid^+^* strain, it also reduced LacZ units in the *lacI^+^Oid^–^* and *lacI^–^Oid^+^* control strains. This effect was also seen (Figure 3G) when low levels of expression of 186 CI were used to avoid potential cellular effects by the use of the pCYMR-4 variant, which uses a weaker expression promoter (Supplementary Figure S3). Expression of 186 CI also inhibited LacZ activity in reporters that contain non-186 sequences at sites 1 and 2, suggesting that 186 CI is somehow affecting LacI expression or its repression of *Plac*. The cause of this effect is currently unresolved and prevents conclusions about 186 CI DNA looping from the assay.

We also attempted to test looping by the phage P1 RepA DNA replicase protein, which has been proposed to interact when bound to iteron sequences located on different DNA molecules in a ‘handcuffing’ interaction that is thought to help control the copy number of the plasmid P1 prophage (8). However, we found that the expression of P1 RepA also affected LacZ activity of the control strains (Figure 3H).

These results show that the loopometer is not a foolproof assay for DNA looping and highlight the importance of using the control reporter strains.

It has been pointed out to us that these control strains would not reveal the effects of an expressed protein on general factors that could specifically affect DNA looping, such as levels of DNA supercoiling (65) or nucleoid-associated proteins (21,66). In cases where such effects are suspected, we recommend testing the effect of the expressed protein on *Oid^+^* and *Oid^–^* reporters that do not contain binding sites for the protein, in order to detect any confounding effects on LacI looping.

### Model-based quantitation of looping strength

A simple display of whether or not a candidate protein and its sites interact to loop DNA provides useful information about the mechanism of action of the protein. The fractional reduction in LacZ units in the *lacI^+^Oid^+^* reporter relative to the *lacI^+^Oid^–^* reporter (Figure 3) allows a comparison of the looping effect of the different proteins, sites and concentrations, but does not provide quantitation of the relative strengths of looping. Quantitation of looping strength can provide more information about the effect of concentrations or site variants, the likely importance of looping in the protein’s activity and allows better comparison of DNA looping by different proteins.

**Figure 4.**
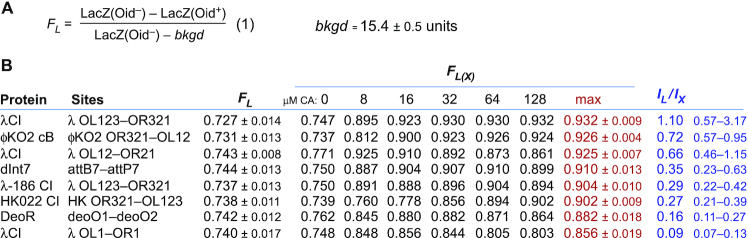
Calculating fractional looping. (**A**) Equation (1) for calculation of fractional LacI looping from reporter LacZ units is from Hao *et al.* (34). Background units were measured with the *lacI^+^Oid^+^ Plac^–^* reporter strain ID1285 (*n* = 16). (**B**) *F_L_* values were calculated from paired *Oid^+^* and *Oid^–^* assays in the absence of the candidate protein (*n* = 12; except *n* = 10 for λCI:*OL1-OR1*; 0 and 128 μM CA values pooled). *F_L(X)_* values for LacI looping in the presence of the candidate protein at each CA concentration were calculated the same way (*n* = 6, except *n* = 5 for λCI:*OL1-OR1*). Values are ±95% confidence limits (Student's *t*). The 95% confidence intervals for the *I_L_/I_X_* values were determined numerically from 1000 calculations of *I_L_/I_X_* in which the values for *F_L(X)_*, *p*, *q* and *r* were individually varied according to their errors.

To quantitate looping strength, the first step is to calculate the fractional LacI looping *F_L_*, that is, the fraction of time that *Oid* and *O2* are looped by LacI. This is obtained using Equation (1) (Figure 4A; (34)), which takes into account background LacZ units that result from low level *lacZ* expression from sources other than *Plac*. This background can be measured using strain ID1285, which carries a mutated *Plac* promoter (‘Materials and Methods’ section), giving ∼15 LacZ units with our assay. The fractional decrease in units seen with the *lacI^+^Oid^+^* reporter relative to the *lacI^+^Oid^–^* reporter gives a linear read-out of the fractional looping; when there is no looping, the *Oid^+^* and *Oid^–^* units are the same, giving *F_L_* = 0; if looping were 100%, *Plac* would be fully repressed and the *Oid^+^* units would equal the background, giving *F_L_* = 1.

Figure 4B shows the *F_L_* values calculated from the data of Figures 2 and 3. In the absence of the test proteins, the *lacI^+^Oid^–^* and *lacI^+^Oid^+^* reporters give *F_L_* values (for LacI alone) of ∼0.74. That is, at the LacI concentration in our reporters, the 3 kb loop between *Oid* and *O2* is formed ∼74% of the time. *F_L(X)_*, the fraction of LacI looping in the presence of the various internal looping proteins, is increased over these *F_L_* values (Figure 4B). Slight increases in looping are seen with uninduced expression (0 μM CA) due to some leak in CymR repression. The maximal values of *F_L(X)_* allow the looping strengths of the test proteins and sites to be ranked. In this set, the strongest looping was exhibited by λCI/*OL123-OR123* and the weakest looping by λCI/*OL1-OR1*, with the other proteins and sites giving intermediate looping strengths. We note that this comparison between different proteins is based on the assumption that differences in the structure of the bridge formed by the different proteins between sites 1 and 2 do not substantially affect LacI looping, that is, that the measured differences in LacI looping are due solely to differences in the *frequency* of formation of the internal loop. The large size of the external loop, 500 + 500 bp, gives us some confidence in this assumption.

The *F_L_* and *F_L(X)_* values can be used to move beyond a simple ranking and to obtain estimates of the relative looping strengths by estimating the strength of looping relative to LacI.

**Figure 5.**
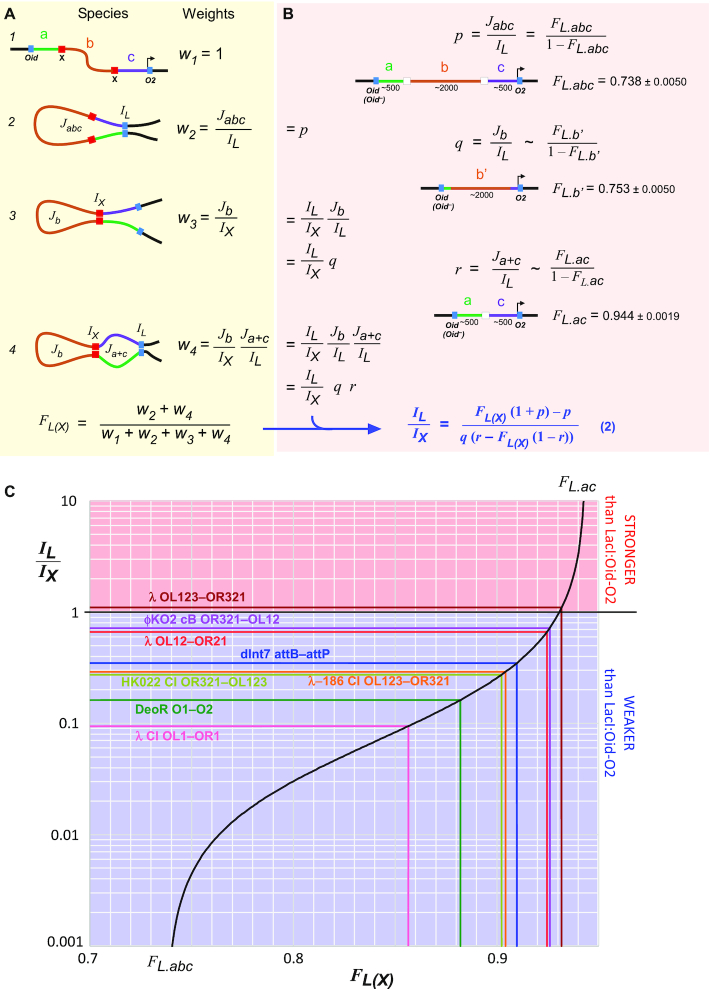
Estimating looping strength relative to LacI/*Oid-O2*. (**A**) Our statistical–mechanical model for two nested DNA loops applied to the case of the loopometer reporter (see text). (**B**) Derivation of Equation (2), which relates *I_L_/I_X_* (the looping strength of the candidate protein and sites relative to LacI:*Oid-O2*) to loopometer measurements of *F_L_* and *F_L(X)_* and the statistical weights *p*, *q* and *r* for looping of particular DNA segments by LacI alone (see text). *F_L.abc_* is the mean of the eight *F_L_* values in Figure 4 (*n* = 8), but values for individual reporters could also be used. *F_L.b__’_* and *F_L.ac_* values (*n* = 32 and 28) were obtained from strains ID1290 and ID1291 or ID1292 and ID1293, respectively. Values are ± 95% confidence limits (Student's *t*). (**C**) The plot of Equation (2), using the *p*, *q* and *r* values of (B), showing the measured *F_L(X)_* values and derived *I_L_/I_X_* values of the proteins/sites tested.

The approach uses a simple model for loop assistance (Figure 5A; 35). The model specifies four species due to the looped or nonlooped state of each pair of sites. Each of these species can be assigned a statistical weight or relative propensity (*w_1_* to *w_4_*). The propensity to form a DNA loop is a balance between the energetic *cost* of bringing together two DNA sites (primarily entropic at these long distances), and the energetic *benefit* provided by the interaction of the sites due to the protein–DNA and protein–protein interactions involved. The cost is inversely related to the effective relative concentration of the two DNA sites, *J*, which for sites in *cis* is a function of the distance between them along the DNA (29,35,36). We represent the benefit by the factor *I*, which is effectively a loop dissociation constant, being inversely related to the benefit and having units of concentration (35). *I* quantitates the ‘looping strength’ of a protein and its DNA sites, with lower *I* indicating higher looping strength. *I* is determined in a complex way by the specific DNA:protein and protein:protein binding constants, the protein’s concentration and the various looping and nonlooping complexes formed at the two DNA sites. Each of the weights for the single-looped species is given by the ratio of the *J* factor for the DNA loop and the *I* factor for the protein-mediated pairing (Figure 5A). The weight for the double-looped species is the weight for the internal loop (*w_3_*) multiplied by a weight representing the closure by LacI of the small loop comprising the a and c arms bridged by the candidate protein (Figure 5A; 35). The fractional LacI looping in the presence of the candidate protein, *F_L(X)_*, is then the sum of the weights for the LacI looped species divided by the sum of all weights (Figure 5A).

Using the loopometer data and two additional measurements, it is possible with this model to obtain an estimate of the ratio of the *I* value for the candidate protein (*I*_X_) relative to that of LacI between *Oid* and *O2* (*I*_L_), *I*_L_/*I*_X_. The weights *w_2_* to *w_4_* can be expressed in terms of *I*_L_/*I*_X_ and three weights for LacI looping of the various DNA segments: *J_L.abc_/I_L_*, *J_L.b_/I_L_* and *J_L.a+c_/I_L_*, designated *p*, *q* and *r* for brevity (Figure 5B). Substituting these terms into the equation for *F_L(X)_* allows *I*_L_/*I*_X_ to be obtained from *F_L(X)_* if *p*, *q* and *r* are known (Figure 5B, Equation 2). Estimates of these three weights can be obtained because the weight for a single DNA loop is related to *F* for that loop: *J*/*I* = *F*/(1–*F*) (35). The weight *p* can be obtained from the *F_L_* measurement of LacI looping of the loopometer in the absence of the internal protein (Figure 5B). To estimate *q*, we constructed and assayed *Oid^+^* and *Oid^–^* versions of reporters with *Oid* and *O2* separated by a 2 kb b-like DNA segment (Figure 5B; strains ID1290 *lacI^+^Oid^+^* and ID1291 *lacI^+^Oid^–^*; ‘Materials and Methods’ section ). This b’ segment contains most of the internal loopometer b segment, but with different sequences near *Oid* and *Plac.O2*. Measurement of LacI looping in these reporters measures *F_L.b__’_*, giving an estimate for *q = J_b_/I_L_*. We note that equal looping of these b’ and b sequences by LacI has not been demonstrated. To estimate *r*, we constructed and assayed reporters in which the internal b segment was removed, leaving *Oid* and *O2* separated by a 1 kb ac segment comprised of the joined 500 bp a and c arms (strains ID1292 *lacI^+^Oid^+^* and ID1293 *lacI^+^Oid^–^*), allowing measurement of *F*_L.ac_. Assuming that the looping of the DNA-joined ac segment is similar to the looping of the a+c segments when they are brought together by the b-looping bridge formed by the candidate protein, we can use *F_L.ac_* as a proxy for *F_L.a+c_*. We note that we saw some divergence from this assumption in a previous study, suggesting that the internal b loop or the protein bridge may affect the looping of the a and c arms (35). Thus, the *F_L_* and *F_L(X)_* measurements from the loopometer, combined with assays of four pre-made strains allows estimation of *I_L_/I_X_* for the candidate protein and its sites.

The *I_L_/I_X_* values calculated in this way for the tested looping proteins and their sites are given in Figure 4B. Note that higher *I*_L_/*I*_X_ values indicate higher looping strength of the candidate protein relative to LacI/*Oid-O2* (as *I* is inversely related to the interaction strength). The relationship between *I_L_/I_X_* and *F_L(X)_* is plotted in Figure 5C, allowing a simple read-out of *I_L_/I_X_* from the loopometer measurement of *F_L(X)_*. The various errors in the measurements result in substantial uncertainties in these *I_L_/I_X_* values, particularly for the stronger looping proteins. For λCI looping between *OL123-OR123*, we obtained *I_L_/I_X_* = 1.1, with a 95% confidence interval of 0.57–3.17 (Figure 4B). This range spans the *I_L_/I_X_* value of 2.7 obtained previously by our more direct comparison of λCI/*OL123-OR123* and LacI/*Oid-O2* looping (35). The steepness of the *I_L_/I_X_* versus *F_L(X)_* plot at high *F_L(X)_* values indicates that the loopometer is not well suited for distinguishing between very strong DNA-looping proteins. However, the curve shows that the loopometer should be capable of detecting and measuring very weak looping, down to looping ∼100-fold weaker than LacI/*Oid-O2*.

The *I_L_/I_X_* values do not provide absolute measurements of looping strength for individual proteins, but the internal LacI standard allows the looping strengths of the different proteins to be quantitatively compared. For example, the data indicate that maximal looping by λCI between *OL12* and *OR12* is some 2-fold weaker than for the full 3-operator sites, with single operator *OL1-OR1* looping a further 5-fold weaker. Importantly, the use of these *I_L_/I_X_* values should assist the comparison of looping strengths of proteins that may be assayed under different conditions, aiding comparisons between assays done in different labs.

**Figure 6.**
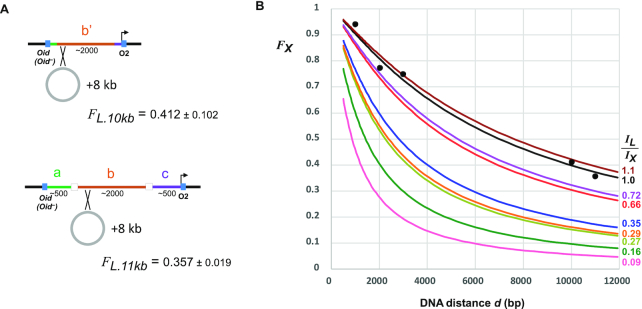
Estimating fractional looping for different DNA distances. (**A**) Reporters with increased *Oid-Plac.O2* spacings were made by integrating an 8 kb plasmid into the abc and b’ segments of reporter strains to make strains ID1318/ID1319 (abc: *Oid^–^*/*Oid^+^*) and strains ID1320/ID1321 (b’: *Oid^–^*/*Oid^+^*). *F_L_* values (± 95% confidence limits, Student's *t*, *n* = 8) were measured as in Figure 4. (**B**) Plot of predicted fractional looping by the candidate protein, *F_X_*, versus DNA distance, *d* (bp), for different values of *I_L_/I_X_*, using the relationships *F* = *J*/(*J*+*I*) (35) and *J* = 1.04 × 10^6^ × *d*^1.15^ nM (36) and assuming *I_L_* = 38.3 nM. Points are *F_L_* looping values for LacI/*Oid-O2* from Figure 5 and (A) and are reasonably fitted by the *F_X_* curve for *I_L_/I_X_* = 1.

Ideally, the measurement of *I_L_/I_X_* in the loopometer would allow estimation of the fractional looping of the protein and its sites at any DNA distance. The fractional looping for a single loop is given by the simple relation: *F* = *J*/(*J* + *I*), and since we previously estimated *I_L_* = 83 nM (35), we should be able to obtain *I_X_* from the *I_L_/I_X_* value. We also determined a power law relationship between *J* and DNA distance *d* (bp): *J* = 1.04 × 10^6^ × *d*^1.15^ nM by measuring LacI looping over distances ranging from 300 to 50 000 bp in the *E. coli* chromosome (summarized in (36)). Together, this should allow the *F* versus *d* relationship to be obtained for any *I_L_/I_X_* value. However, these previous measurements were made with an older version of our LacZ assay, which we now know gives an underestimate of LacI looping (Supplementary Figure S5). To recalibrate the relationship between LacI looping and DNA distance, we constructed loopometer reporters in which the *Oid-O2* distance was increased by 8 kb by the integration of a plasmid into an integrase attachment site that had been included in our original constructs (Figure 6A; ‘Materials and Methods’ section). By assaying *Oid^+^* and *Oid^–^* versions of these reporters, we thus obtained *F_L_* values for ∼10 and 11 kb spacings. Combining these with the *F_L_* measurements for ∼1, 2 and 3 kb spacings (Figure 5B), we found a reasonable match between these five values and the predictions of the *F* versus *d* relationship (Figure 6B; *I_L_/I_X_* = 1 curve), if either *I_L_* was about 2-fold lower, or the power law prefactor was about 2-fold higher, than our previously measured values. This recalibration allows the fractional looping of the proteins and their sites at any DNA distance on the *E. coli* chromosome to be estimated from the *I_L_/I_X_* values (Figure 5). For example, the loopometer measurement of *I_L_/I_X_* = 0.16 for DeoR/deoO_1_-O_2_ predicts ∼72% looping between these sites at their natural 600 bp spacing (65–82% based on the 95% confidence interval for *I_L_/I_X_*) and 20% looping at a 5 kb separation. The predicted looping between λ *OL123* and *OR123* at their natural 2.3 kb spacing is ∼79%, in agreement with an independent measurement using high-resolution live-cell microscopy ((31); though uncertainty in *I_L_/I_X_* gives a large range for predicted *OL123*-*OR123* looping: 48–92%). Note that prediction of looping frequencies for spacings below 500 bp is unreliable due to the onset of helical phasing sensitivity at these distances.

## CONCLUSIONS

The loopometer represents a simple assay that can provide strong evidence for DNA looping *in vivo*. At a minimum, it requires only the construction of two plasmids—one for the binding sites and one to express the candidate protein—followed by sequential integration of these (and the empty expression plasmid) to make six bacterial strains for the LacZ assay. Quantitation of the looping strength relative to LacI/*Oid-O2* is enabled by further assays of five pre-made strains, for measuring background and for LacI looping calibration.

Aside from the detection and measurement of DNA looping, the assay permits examination of the effect of protein concentration on looping, which may be an important factor in regulation. Additional constructions and assays can provide comparisons of looping between different sites, including testing the effect of binding site mutations. Similarly, the effects of protein mutations on looping can readily be examined, though the assay does not by itself distinguish between effects on protein–protein versus protein–DNA interactions.

The *in vivo* fluorescence microscopy-based assay of DNA looping by Hensel *et al.* (31) is the only approach that is currently able to provide similar information to the loopometer. In this study, the *OL-* and *OR*-binding sites for λ CI were placed adjacent to sites bound by fluorescently tagged proteins, and the distances between these sites were measured in live cells in the presence of CI. While this assay could readily be adapted to other proteins, it requires specialized equipment and expertise and is likely to be limited to strong DNA-looping proteins, as only these can efficiently loop DNA over the long distances required to resolve looped and unlooped states microscopically.

The loopometer assay currently has a number of limitations, some of which should be able to be overcome by further development: (i) The assay can fail if there are confounding effects of the expressed protein on looping-independent expression of *lacZ*. This could potentially be overcome by the use of a different reporter or by replacing LacI with a different looping protein. (ii) Comparison of looping strength between different proteins relies on the untested assumption that the precise structure of the bridge formed by the different proteins between sites 1 and 2 does not substantially affect LacI’s ability to loop the two 500 bp DNA arms. We suspect that DNA arms of different lengths could be used to test and validate this assumption. (iii) The assay only tests homotypic interactions, that is looping by a single protein. However, the assay could be easily modified to detect heterotypic interactions by introducing a second protein expression system. (iv) The assay can only test proteins that have specific binding sites and can be expressed in the active form in *E. coli*, which may limit testing of eukaryotic proteins. By itself, the assay does not provide evidence for the activity of the protein unless looping is observed. The use of fusions to bacterial DNA-binding domains (e.g. the λ CI NTD) may extend the range of testable proteins, since the ability to fold an active DNA-binding domain would not be required. We note that such fusions could provide an alternative bacterial one- or two-hybrid assay, allowing detection of interacting domains by DNA looping. (v) A bacterial assay may not be appropriate for eukaryotic looping proteins because of the lack of necessary accessory factors (e.g. nucleosomes). However, the principle of detecting DNA-looping proteins by loop assistance should also work in eukaryotic cells, as studies have shown that DNA-looping interactions can increase gene expression by bringing an enhancer closer to the promoter (67–69). Thus, insertion of a DNA sequence flanked by binding sites for a candidate DNA-looping protein between a promoter and enhancer, combined with an inducible protein expression system, could be used to generate a qualitative eukaryotic loopometer.

## Supplementary Material

gkaa1284_Supplemental_File

## Notes

Present address: Adrienne E. Sullivan, Quantitative Cell Biology Laboratory, The Francis Crick Institute, London, UK.
