# Supplementary material for: The loopometer: a quantitative in vivo assay for DNA-looping proteins
Source: Nucleic Acids Res. 2021 Jan 28;49(7):e39. doi: 10.1093/nar/gkaa1284 (PMC8053113; doi:10.1093/nar/gkaa1284)
Supplement: gkaa1284_Supplemental_File [file gkaa1284_supplemental_file.pdf]

Supplementary Data

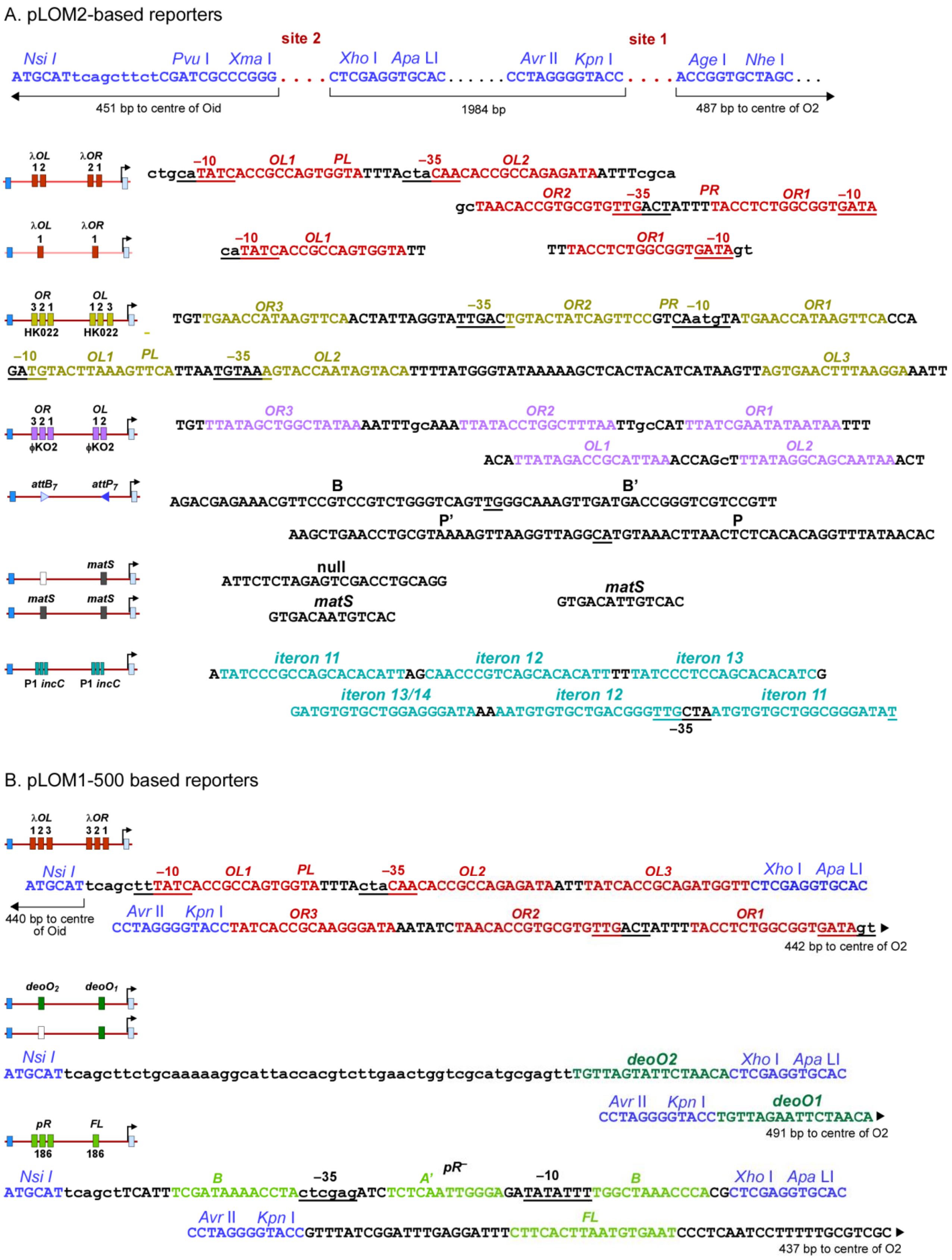

Fig S1. Protein binding sequences

Sequences inserted into sites 1 and 2. Lowercase bases are changes from native sequences introduced to reduce promoter activity. Underlined bases are promoter elements and the central bases of the *att*$_7$ sites

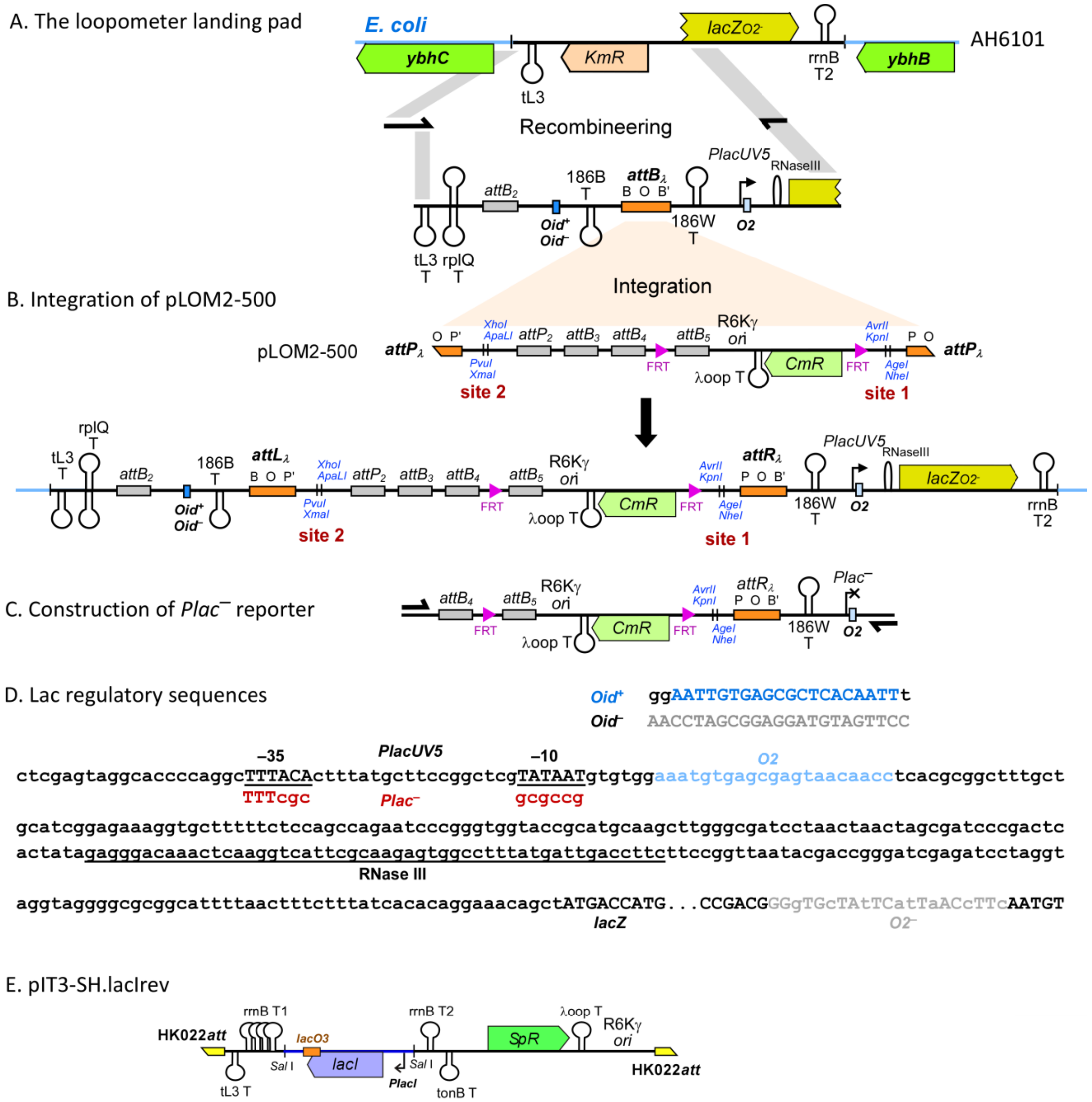

Fig S2. Reporter constructions

A. Construction of the loopometer landing pad by replacement of part of a *KmR*.'lacZ cassette that had replaced *attB*$_{\lambda}$ in E4643 (MG1655 junction sequences ACTGAAAATGTGTTC 807279 and 807391 TTTAAGTGATACCAG), with an *Oid*$^{+}$/*Oid*$^{-}$.*attB*$_{\lambda}$.*PlacUV5.O2.lacZ'* PCR fragment. Stem loops are transcription terminators.

B. Structure of pLOM2-500 and the landing pad after its integration at *attB*$_{\lambda}$. *att* sites 2-5 are from the collection of Yang et al. (44).

C. PCR fragment used to insert the *Plac*$^{-}$ promoter into a reporter with the *FRT-FRT* segment removed.

D. Sequences of LacI regulatory elements and the *lacZ* leader.

E. Structure of the plasmid integrated at *attB*$_{HK022}$ to supply a constant level of LacI

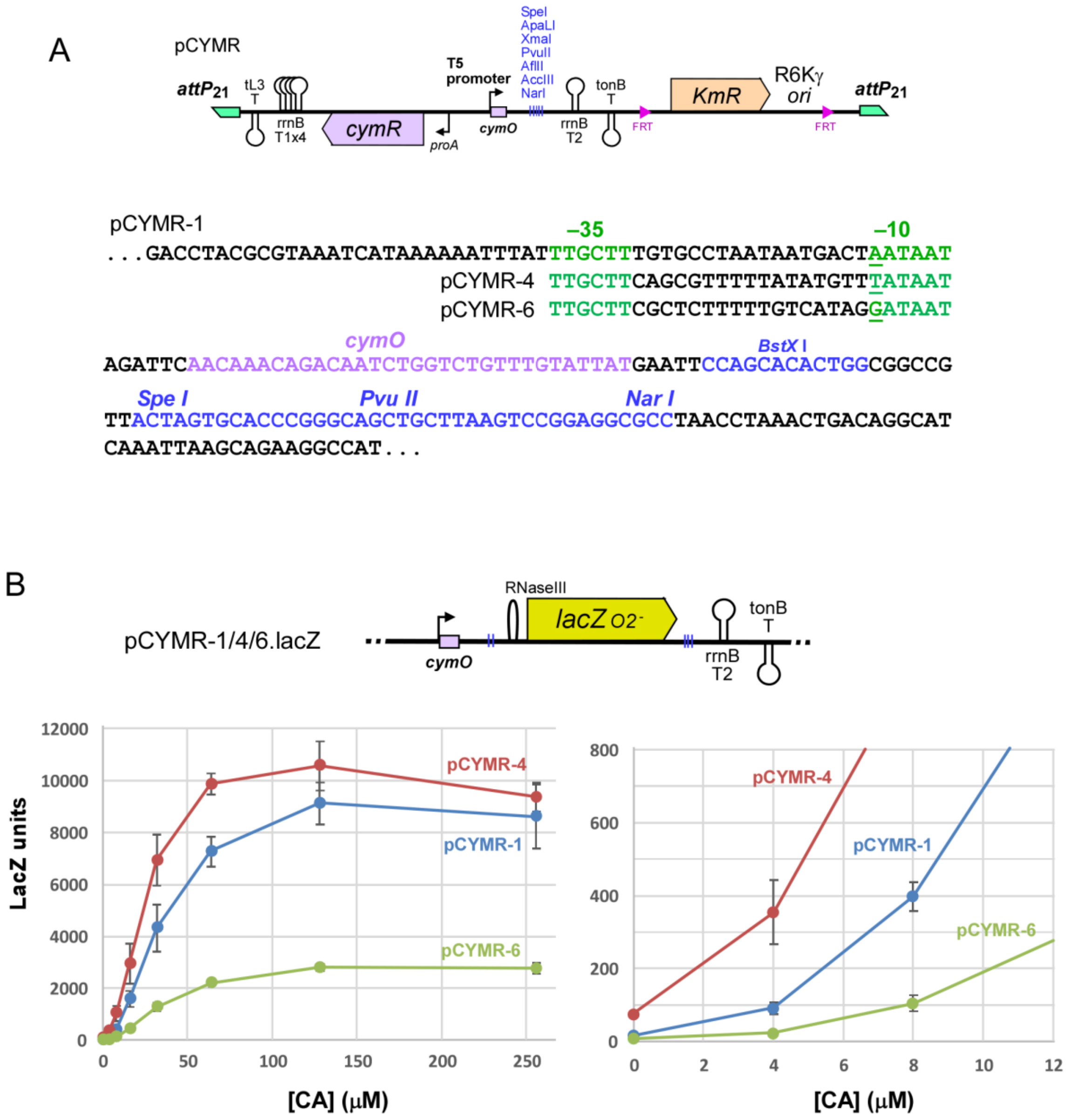

Fig S3. The pCYMR expression vectors

A. Structure of the pCYMR vectors and sequences around the promoter and cloning site in the three promoter variants. The differences in the –10 sequences are underlined.

B. Expression was tested by inserting *lacZ* into the three variant plasmids, integrating in single copy, and inducing with cumic acid (CA).

BstX I Spe I Apa LI Xma I Pvu II Afl III Acc III Nar I

...CCAGCACACTGGCGGCCGTTACTAGTGCACCCGGGCAGCTGCTTAAGTCCGGAGGCGCC...

pCYMR-1.λcI

Apa LI M S T K K ... E E T F G * Nar I

GTGCACcccttgcggtgatagatttaacgtATGAGCACAAAAAAG...GAAGAGACGTTTGGCTGAGGCGCC

λOR3

pCYMR-1.λ-186cI

Apa LI M S T K K ... Y S E V N * Nar I

GTGCACcccttgcggtgatagatttaacgtATGAGCACAAAAAAG...TACAGCGAGGTTAACTAAGGCGCC

λOR3

pCYMR-1.deoR

Pvu II M E T R R ... I K L M Y * Pvu II

CAGCTGaataactattcagagggattATGGAAACACGTCGC...ATTAAGTTGATGTATTAACAGCTG

pCYMR-1.ϕKO2cB

Pvu II M I N R M ... M V E G R *

CAGCTGgctataaacagatcgtttATGATTAATCGTATG...ATGGTAGAGGGTAGATGACAGCTG

pCYMR-1.HK022cI

Apa LI M V Q Q K ... Y K R F G * Nar I

GTGCACtacctaatatagttgaacttATGGTTCAACAGAAA...TACAAGCGATTCGGATAAaaataaGGCGCC

PM –10

pCYMR-1.int7S10A

BstX I

CCAGCACACTGGCGGCCGTTAgagatc...

M K V A I Y V R V A T D ... D Y T L K * Apa LI

...taaagaggagaaaattcgaATGAAAGTGGCCATTTATGTTCGTGTTgcCACCGAT...GATTACACCCTGAAATAACTAGTGCAC

pCYMR-6.186cI

Apa LI M R I D S ... Y S E V N * Nar I

GTGCACactagaaataattttgtgtaaataggttttatcgaaATGAGAATAGATTCT...TACAGCGAGGTTAACTAAGGCGCC

pCYMR-1.P1repA

Xma I M N Q S F ... L K L P E * Afl III

CCCGGGCAGctgactatccacacaattcggaaaaagtaatATGAATCAATCATTT...CTGAAACTCCCCGAATAAcagCTGCTTAAG

pCYMR-#.lacZ

Spe I' RNaseIII

ACTAGagtcgacctgcaggcatgcaagcttgggcgatcctaactaactagcgatcccgactcactatagagggacaaactcaaggtcattcg...

...caagagtggcctttatgattgaccttcttccggttaatacgaccgggatcgagatcctaggtaggtaggggcgcggcattt...

M T M I T ... V W C Q K * Nar I

...taactttctttatcacacaggaaacagctATGACCATGATTACG...GTCTGGTGTCAAAAATAAtaataacgggcaggccGGCGCC

Fig S4. Genes cloned in pCYMR expression vectors

Introduced leader sequences are in lowercase.

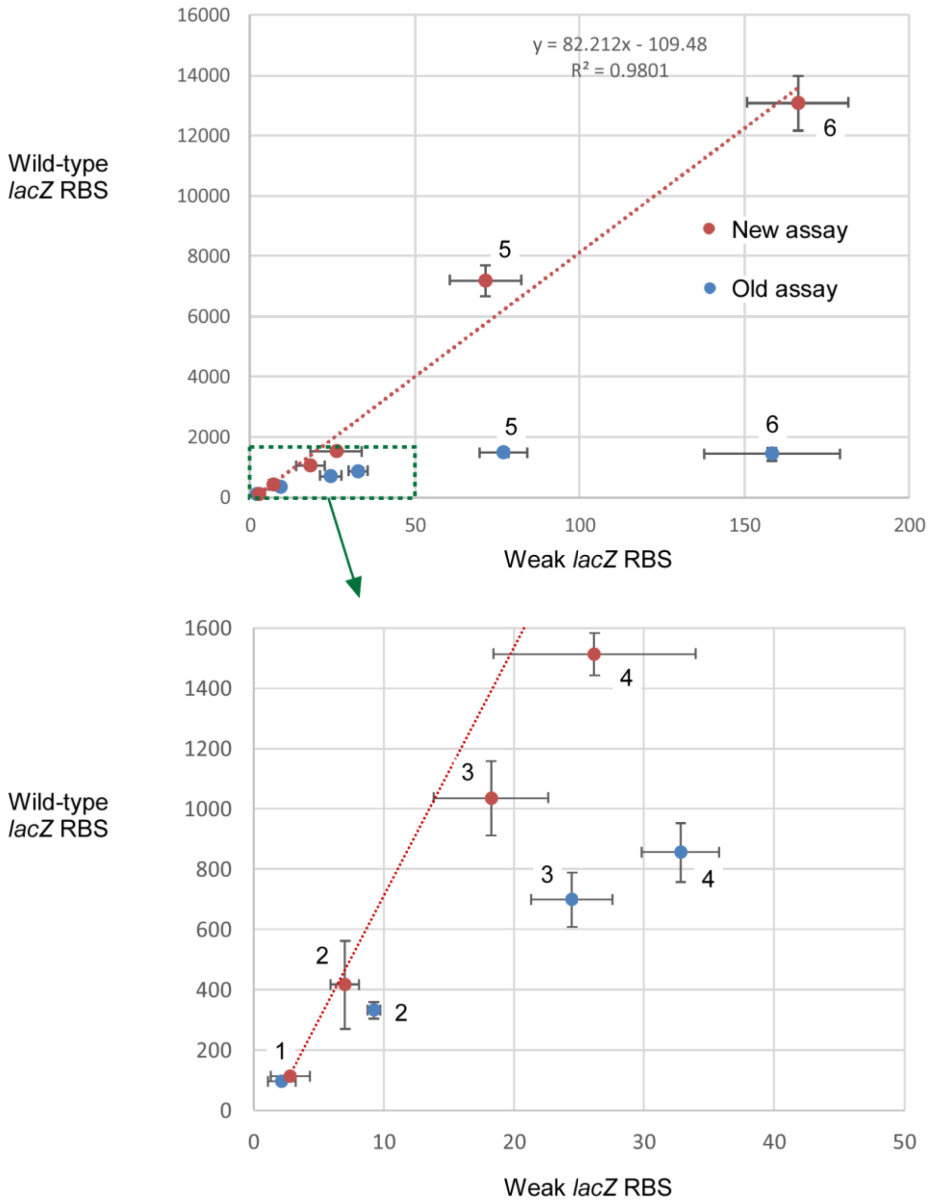

Fig S5. Testing the new lacZ assay

In a previous study, we found that our existing LacZ assay method, which uses only polymyxin B to permeabilize cells, underestimates high LacZ activities (41). This was revealed by assaying *lacZ* reporters carrying the same series of promoters but with different strength ribosome binding sites (RBS) for translation of *lacZ* mRNA. We found that the units for the reporters with the native *lacZ* RBS sequence saturated for stronger promoters relative to the units for the reporters carrying a mutated, weaker *lacZ* RBS sequence (41). Our new LacZ assay (Materials and Methods) includes lysozyme to improve permeabilization, as well as some other changes. To check that the new assay does not suffer from this saturation over the range of promoter activities in the loopometer, we performed old and new assays using 6 pairs of strains that carry the same promoter (promoters 1-6) with either the wild-type *lacZ* RBS or the weaker RBS. The strains are previously described (41), and in order from weakest to strongest promoter (wild-type/weaker RBS) are: promoter 1 - AH2101/AH2140, promoter 2 - AH2124/AH2147, promoter 3 - AH2143/AH2155, promoter 4 - AH2144/AH2146, promoter 5 - AH2107/AH2150, promoter 6 - AH2138/AH2145.

The plots show the LacZ units obtained for the wild-type or weaker RBS reporters for each promoter (error bars are 95% confidence limits, *n*=4, Student's *t*). The plot for the old assay shows the saturation effect, with the LacZ units for the wild-type RBS reporters not proportional to the units for the weaker RBS reporters. In contrast, the plot for the new assay gives a reasonable fit to a straight line, indicating a lack of saturation of the assay up to 13000 LacZ units. The *PlacUV5* promoter used in the loopometer gives ~3000 units.

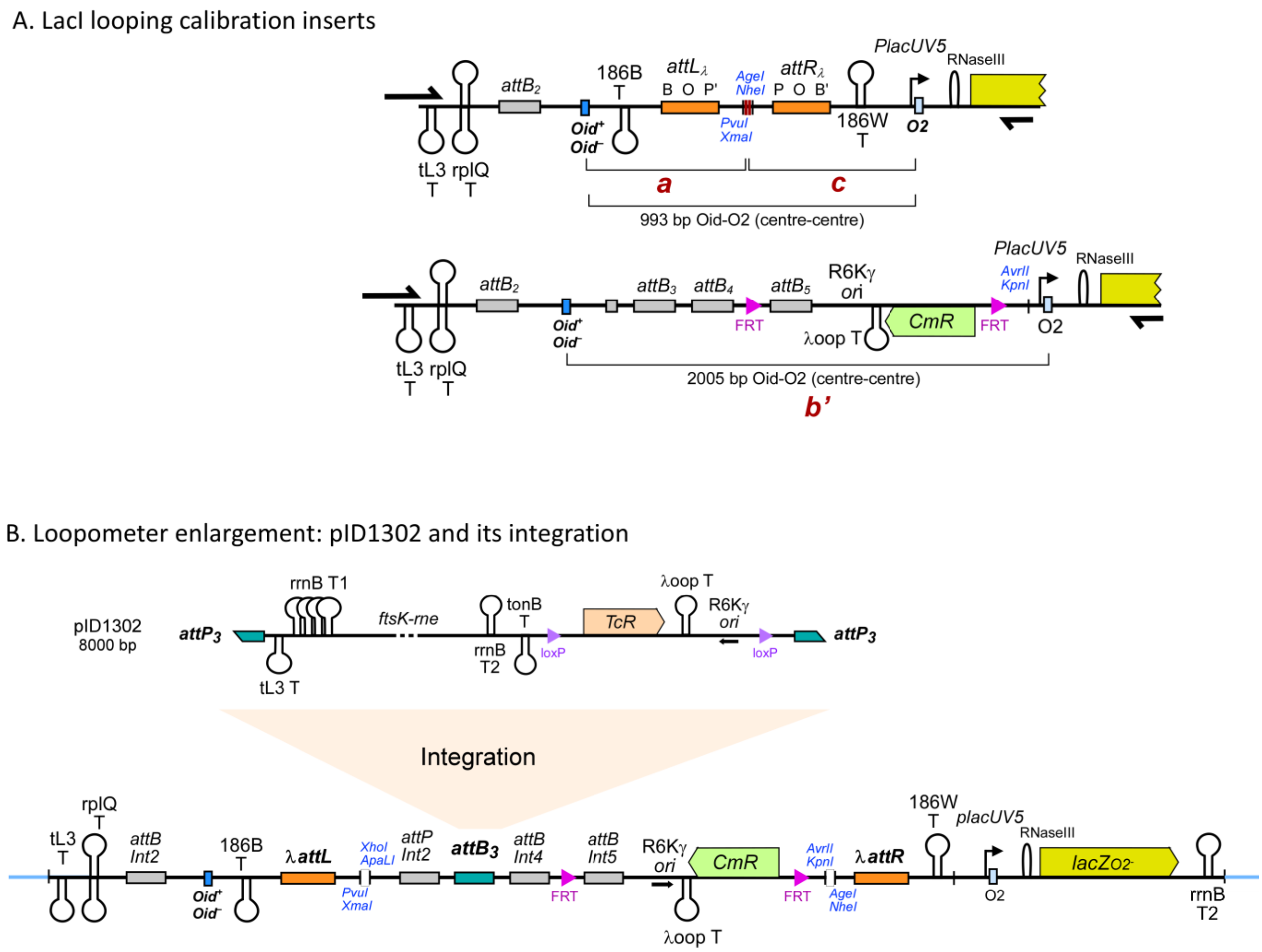

Fig S6. Loopometer calibration constructs

A. PCR fragments used to replace the KmR.'*lacZ* cassette in AH6101 to create the ac and b' reporters of Fig 5A.

B. Structure of pID1302 used to increase the *Oid-O2* distance. Integration was mediated by Int3 (44) expressed from a helper plasmid.
